# A multi-centre randomised controlled trial comparing megestrol acetate to levonorgestrel-intrauterine system in fertility sparing treatment of atypical endometrial hyperplasia

**DOI:** 10.1007/s10815-024-03172-z

**Published:** 2024-08-31

**Authors:** Charissa Shu Ying Goh, Michelle Jia Min Loh, Whui Whui Lim, Joella Xiahong Ang, Ravichandran Nadarajah, Tze Tein Yong, Pearl Tong, Yen Ching Yeo, Jessie Wai Leng Phoon

**Affiliations:** 1 Department of Reproductive Medicine, KK Women’s and Children’s Hospital, 100 Bukit Timah Road, Singapore, 229899 Singapore; 2 Department of Obstetrics & Gynaecology, Singapore General Hospital, Singapore, Singapore; 3 Division of Gynaecologic Oncology, National University Hospital, Singapore, Singapore; 4 Department of Pathology and Laboratory Medicine, KK Women’s and Children’s Hospital, Singapore, Singapore

**Keywords:** Atypical endometrial hyperplasia, Fertility, Megestrol acetate, Levonorgestrel-intrauterine system, Randomised controlled trial

## Abstract

**Purpose:**

The objective of the trial was to compare the regression rate of atypical endometrial hyperplasia (AEH) in patients treated with megestrol acetate (MA) vs. levonorgestrel-intrauterine device (LNG-IUS). We also aimed to assess the fertility and pregnancy outcomes in these patients.

**Methods:**

The study was a phase II multi-centre randomised controlled trial on the use of MA compared to LNG-IUS in the treatment of AEH conducted from January 2020 to January 2024 in Singapore. Women who were diagnosed with AEH and between 21 and 40 years old were included. The patients were randomised to receive either MA (160 mg orally daily) or LNG-IUS. The primary outcomes assessed were the regression rates at 3 months, 6 months and 9 months of treatment. The secondary outcomes assessed were the side effects, patient acceptability and fertility outcomes.

**Results:**

Thirty-six patients completed the trial. The overall regression rate was 88.9% by 9 months. There was no statistically significant difference in the 9-month complete regression rate between MA vs. LNG-IUS. There was also no significant difference in side effects and weight change between both arms. Nineteen patients were actively pursuing fertility after complete regression. There were 8 pregnancies achieved, with resultant 4 live births and 4 miscarriages.

**Conclusion:**

Our study confirms a high regression rate of AH with medical treatment. LNG-IUS is a non-inferior treatment compared to megestrol acetate. Successful pregnancy outcomes can be achieved after regression of AEH. Long-term studies of sufficient sample-size are needed to assess for fertility and pregnancy outcomes, risk of recurrence and long-term risk of malignancy.

**Trial registration number:**

The study was registered with the Health Science Authority (HSA) (License No.: CTA1900087) on September 5, 2019: https://eservice.hsa.gov.sg/prism/ct_r/enquiry.do?action=loadSpecificDetail.

The trial was registered retrospectively on ClinicalTrials.gov (ID: NCT05492487) on April 7, 2022: https://clinicaltrials.gov/study/NCT05492487.

## Introduction

Endometrial hyperplasia is a growing clinical problem that has implications on oncology and fertility outcomes. The risk of progression of atypical endometrial hyperplasia (AEH) to cancer is up to 30% [1]. As such, the gold standard treatment of AEH is hysterectomy. However, approximately 5% of patients diagnosed with AEH will be under 40 years [2]. Endometrial cancer is the 5th most prevalent worldwide, and the 4th most prevalent cancer in women from high-income countries including Singapore [3]. Globally, there is rise in obesity and metabolic diseases. As these are significant risk factors for the development of endometrial hyperplasia and cancer, the prevalence of endometrial hyperplasia and cancer is likewise on the rise [4, 5]. With the global trend for delayed marriage and delayed childbearing age, these women are increasingly opting for fertility conservation treatment.

Different medical therapies have been used to treat endometrial hyperplasia. These therapies often include progesterone in the form of oral progestins and/or progesterone intrauterine devices [6–14]. Metformin has also been used as an adjunct treatment, but its efficacy and target treatment population are still being debated upon [11, 15].

A Cochrane review [6] published in 2017 found that there were “no randomised controlled trials (RCTs) of women with atypical endometrial hyperplasia” and the findings were derived from a subgroup of 19 women in a larger RCT [7]. The review concluded that there was insufficient evidence to draw any conclusions regarding the relative efficacy of levonorgestrel-intrauterine system (LNG-IUS) versus oral progesterone in this group of women [6]. To-date, there remains limited high-quality data on the efficacy on type of medical treatment of atypical endometrial hyperplasia. From our review of the current literature, there is only one RCT by Xu Z et al. comparing the use of megestrol acetate (MA) vs. LNG-IUS vs. MA and LNG-IUS in women with AEH [8]. The trial found no difference in complete regression rate between the megestrol acetate (MA), the levonorgestrel intra-uterine system (LNG-IUS) and the combination of MA + LNG-IUS arms [8].

Prospective data on pregnancy and live birth outcomes of the type of medical treatment of AEH is limited to the RCT by Xu Z et al. [8]. A Cochrane review is underway to evaluate the regression and live birth rates in this group of women [16]. Retrospective data in literature has reported successful pregnancies in women who undergo fertility-sparing treatment for atypical hyperplasia, with pregnancy rates ranging from 56 to 63% in women treated with oral progestins or LNG-IUS respectively [17]. The follow-up of patients on medical treatment for AEH requires frequent intrauterine operations, including dilation and curettage (D&C) and endometrial biopsy. These interventions can theoretically increase the risk of endometritis, and cause endometrial thinning or intrauterine adhesions, which in turn, can adversely affect implantation and pregnancy development. D&C as a method of endometrial biopsy during follow-up has been shown to be possibly associated with a decrease in endometrial thickness during ovulation [18], an increase in risk of placenta accreta [19] and lower pregnancy rates [17], although the evidence is not conclusive. We hypothesise that a shorter time to regression (and therefore, less repeated hysteroscopy and biopsies) may be associated with better fertility outcomes.

Regarding side effects, LNG-IUS has been shown to be associated with higher incidence of irregular bleeding [6]. On the other hand, MA is associated with increased appetite and weight gain. A retrospective study by Park JY et al. [20] found that women who received megestrol for early-stage endometrial cancer had an increase in weight after treatment. However, weight change during therapy was not significantly associated with complete response, recurrence rate, pregnancy or live birth rates. There was no comparison with alternatives such as LNG-IUS.

The aim of our trial was to determine if LNG-IUS was non-inferior to MA in the treatment of AEH. We also aimed to determine the side effects, patient acceptability and fertility outcomes in patients with AEH receiving either LNG-IUS or MA.

## Materials and methods

### Study design and patients

The study was a multi-centre, open-label, randomised controlled phase II trial (NCT05492487) with two study arms comparing MA to LNG-IUS. Patients were recruited from January 3, 2020 to July 14, 2023, and were on follow-up from January 3, 2020 to January 8, 2024. The trial was conducted in the three medical centres in Singapore: KK Women’s and Children’s Hospital, Singapore General Hospital and National University Hospital. This study was supported by the Singhealth AM grant (Grant No.: AM/CT004/2020).

Eligible patients with AEH who were aged of 21–40 years and had strong desires to preserve fertility were included in the study. Patients with previous or current history of endometrial cancer, or who were already on treatment for AEH were excluded. Patients who were unable to be randomised (i.e., contraindications to either MA or LNG-IUS) were excluded.

The study was approved by the Singhealth Centralised Institutional Review Board (CIRB) (CIRB Ref: 2019/2551) and registered with the Health Science Authority (HSA) (License No.: CTA1900087) (https://eservice.hsa.gov.sg/prism/ct_r/enquiry.do?action=loadSpecificDetail) on September 5, 2019, prior to the recruitment of the first patient, as per our national requirement. The trial was registered retrospectively on ClinicalTrials.gov (ID: NCT05492487) on April 7, 2022 (https://clinicaltrials.gov/study/NCT05492487). This trial was only registered retrospectively on ClinicalTrials.gov when the study team found out about the need for registration on an approved registry as a requirement for publication. There were no changes to protocol our endpoints during the entire conduct of the study. All patients were fully informed of the benefits and risks of this clinical trial and provided written informed consent.

### Randomisation and masking

Patients were allocated (1:1) to one of two treatment arms: MA vs. LNG-IUS using randomisation envelopes. A total of 60 randomisation envelopes divided over the 3 institutions were created at the start of the trial. The allocation sequence was concealed from the study team enrolling and assessing participants in opaque and sealed envelopes. The study was open labelled, and all patients and study physicians were aware of the treatment assignment.

### Procedures

In literature, the dose of megestrol acetate used for the treatment of atypical endometrial hyperplasia and/or endometrial adenocarcinoma was 160–320 mg per day [8, 21]. In the latest ESGO/ESHRE/ESGE guidelines on fertility-sparing treatment of endometrial cancer, the recommended dose was-320 mg per day [22]. As our study group involved atypical endometrial hyperplasia, we used the starting dose of 160 mg per day.

The patients in the MA arm received continuous oral megestrol acetate 160 mg once daily and the patients in the LNG-IUS arm underwent LNG-IUS (containing LNG 52 mg) insertion.

All patients underwent a hysteroscopy D&C for the first diagnosis before joining the trial to exclude the presence of concomitant endometrial cancer. Pathologic diagnosis was confirmed by the pathologists in each institution, according to the World Health Organization (WHO) pathological classification (2014) [23].

Endometrial evaluations were performed every 3 months to evaluate treatment response after initiation of the treatment until complete response (CR), by a member of the study team. Endometrial evaluation was done by hysteroscopy and targeted endometrial biopsies, or by outpatient endometrial biopsy (without hysteroscopy). The LNG-IUS was kept in situ during the biopsy. If dislodged during the biopsy, a new LNG-IUS would be inserted.

Treatment response was categorized as follows: (1) complete regression (CR): histology showing resolution of endometrial hyperplasia; (2) persistent hyperplasia (PH): persistent endometrial hyperplasia (non-atypical or atypical); (3) progression disease (PD): progression of disease to endometrial cancer. The treatment was continued until CR was achieved, or at a maximum duration of treatment of 9 months in the trial. Treatment was discontinued when patients experienced unacceptable side effects or when patients were keen to switch to another medical treatment or surgery (hysterectomy). Patients with PD would be referred to the gynaecologic oncologist for further management. Patients treated beyond 9 months would exit the study. The decision to continue medical treatment or opt for hysterectomy would be discussed in this group of patients.

Patients in the trial who achieved CR and were keen to achieve fertility would be reviewed by the reproductive medicine unit. Fertility options including ovulation induction, intrauterine insemination with or without superovulation, and in vitro fertilisation would be discussed.

For CR patients with no immediate plans for conception, patients were offered cyclical dydrogesterone, oral contraceptive pills, or insertion of LNG-IUS for prevention of disease recurrence.

Ultrasonography, physical review and/or endometrial biopsies would be performed every 6 months to 1 year to assess for recurrence.

Data on age, presenting complaints, presence of polycystic ovarian syndrome (based on the Rotterdam Criteria), presence of diabetes mellitus, impaired glucose tolerance (IGT) or impaired fasting glucose based on the World Health Organisation (WHO) diagnostic criteria, comorbidities and ultrasonography findings were collected during the study. The patients’ height and weight were collected after every 3 months of treatment (3-month, 6-month and 9-month).

All patients were followed up from the date of treatment initiation to January 8, 2024.

### Outcomes

The primary endpoints were the CR rate at 9 months and the time taken for CR. Secondary endpoints were the side effects, patient acceptability, fertility outcomes (pregnancy, miscarriage and live birth rates) and recurrence.

### Statistical analysis

A previous meta-analysis on retrospective data has shown improved treatment outcome with LNG-IUS compared to oral progestogens [9]: pooled regression of 90% vs. 69% respectively.

The sample size of 60 was calculated to detect a non-inferiority margin difference between the group proportions of − 0.05. The reference group proportion was 0.70. The treatment group proportion is assumed to be 0.65 under the null hypothesis of inferiority. The power was computed for the case when the actual treatment group proportion was 0.90. The test statistic used was the one-sided *Z* test (un-pooled). The significance level of the test was 0.05. Based on retrospective clinical data of AEH patients in our centres, we expected to recruit 60 patients over 2 years. However, due to the finite duration of research funding and the restrictions and difficulty of recruitment during the COVID-19 pandemic, the final recruitment number was 40 patients.

Continuous variables were represented as median and interquartile range, where applicable, and were compared using the Mann–Whitney *U* test. Categorical variables were represented as frequency and percentage, and compared using Pearson’s chi-square test or Fisher’s exact test, where applicable. The significance was set as 2-sided *P* < 0.05. All statistical analyses were performed using Stata 18 (Stata Corporation, USA).

### Role of funding source

The funding body had no role in the study design, data collection, data interpretation, data analysis, or drafting or editing of this manuscript.

## Results

### Patient characteristics

**Fig. 1:**
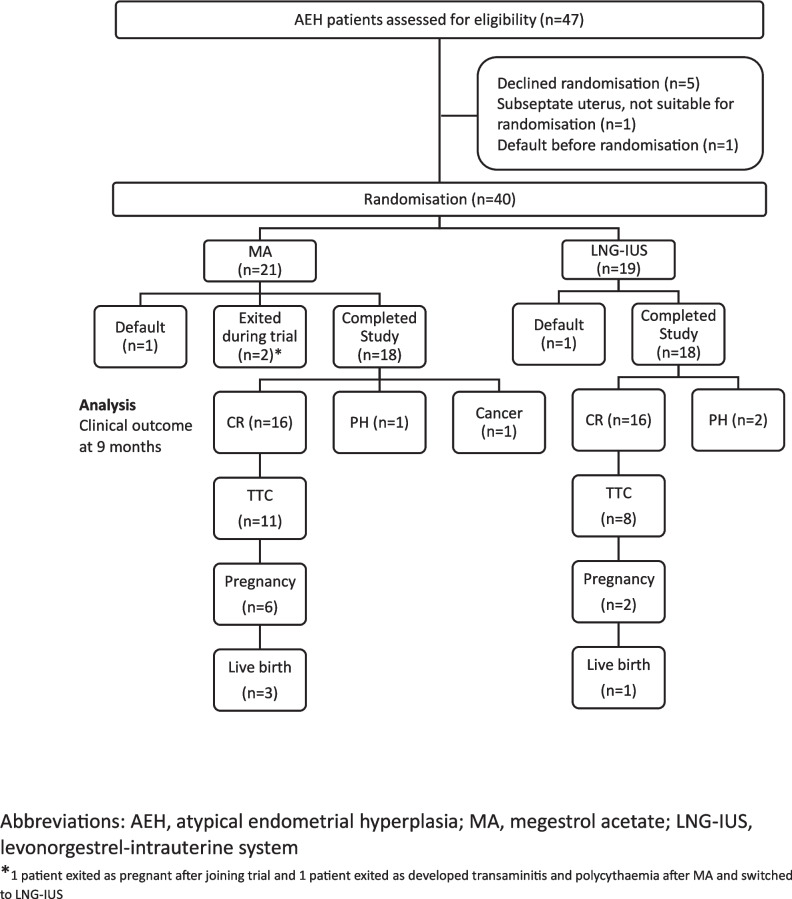
Flowchart of trial patients

The flowchart of patients in the trial is shown in Fig. 1. A total of 47 patients were screened; of them, 5 patients declined randomisation, 1 patient was not suitable for randomisation in view of subseptate uterus which was a contraindication for LNG-IUS, and 1 patient defaulted before randomisation.

Forty patients underwent randomisation (1:1) to MA (*n* = 21) or LNG-IUS (*n* = 19) and 36 patients completed the study (achieved endpoint of CR or PD, or completed 9 months of treatment). There was 1 patient in each arm that defaulted the study during the follow-up. Two patients in the MA arm exited the study before the endpoint was reached.

The patient characteristics are summarised in Table 1. The median age was 32.0 years (range: 23–40 years) and the median BMI was 34.5 kg/m^2^ (range 20.0–55.9 kg/m^2^). Most patients (90.0%) were nulliparous. Eighteen out of 40 (45.0%) of patients had PCOS, 35.0% (14/40) patients had DM, IGT or IFG. The most common presenting complaint was oligomenorrhea (90.0%), followed by abnormal uterine bleeding (62.5%) and subfertility (57.5%). Twenty-six out of 40 patients (65.0%) had a thickened endometrium and/or endometrial polyp noted on ultrasonography.

**Table 1:** Demographic and clinical characteristics of patients recruited into the study

	MA (N = 21)	LNG-IUS (N = 19)	Overall (N = 40)	*p* value
Clinical characteristics				
Age, years				
Median	32.0	32.0	32.0	> 0.950
IQR	30.0–37.0	30.0–37.0	30.0–37.3	
Range	24–40	23–40	23–40	
Weight, kg				
Median	86.1	91.4	87.9	0.401
IQR	73–97.9	78.7–112.4	74.2–101.4	
Range	62.4–141.6	58.0–143.0	58.0–143.0	
BMI, kg/m^2^				
Median	32.8	35.5	34.5	0.185
IQR	29.4–37.0	30.5–44.8	29.9–40.0	
Range	25.1–50.8	20.0–55.9	20.0–55.9	
Nulliparity, *n* (%)	20 (95.2)	16 (84.2)	36 (90.0)	0.331
PCOS, *n* (%)	13 (61.9)	5 (26.3)	18 (45.0)	0.024
DM/IGT/IFG, *n* (%)	7 (33.3)	7 (36.8)	14 (35.0)	0.750
Presenting complaint, *n* (%)				
Oligomenorrhea, *n* (%)	18 (85.7)	18 (94.7)	36 (90.0)	0.607
AUB, *n* (%)	12 (57.1)	13 (68.4)	25 (62.5)	0.462
Subfertility, *n* (%)	12 (57.1)	11 (57.9)	23 (57.5)	> 0.950
Clinical investigation findings, *n* (%)				
Thickened endometrium and/or polyp, *n* (%)	12 (57.1)	14 (73.7)	26 (65.0)	0.273
Baseline Hb, g/dL ± SD (range)	12.9 ± 2.1 (8.1–16.7)	12.4 ± 1.9 (9.2–15.4)	12.7 ± 2.0 (8.1 – 16.7)	0.378

Continuous variables were summarized as medians, IQR and ranges. Categorical variables were presented as frequencies and percentages

Abbreviations: *IQR*, interquartile range; *MA*, megestrol acetate; *LNG-IUS*, levonorgestrel-intrauterine system; *SD*, standard deviation; *BMI*, body mass index; *PCOS*, polycystic ovarian syndrome; *DM*, diabetes mellitus; *IGT*, impaired glucose tolerance; *IFG*, impaired fasting glucose; *AUB*, abnormal uterine bleeding; *Hb*, haemoglobin

### Primary endpoint — CR rates, time taken for regression

The results of the disease outcomes are summarised in Table 2.

**Table 2:** Regression rate and side effects of patients in the trial

	MA (N = 18)	LNG-IUS (N = 18)	Overall (N = 36)	*p* value
Disease outcomes				
Complete Regression				
At 3 months, N (%)	7 (38.9)	11 (61.1)	18 (50.0)	0.182
At 6 months, N (%)	14 (77.8)	15 (83.3)	29 (80.6)	> 0.950
At 9 months, N (%)	16 (88.9)	16 (88.9)	32 (88.9)	> 0.950
Persistence	1 (5.6)	2 (11.1)	3 (8.3)	> 0.950
Cancer	1 (5.6)	0	1 (2.8)	> 0.950
Side effects				
Weight change				
Median weight change, kg (IQR)	+ 0.5 ((− 2.3)-3.7)	+ 0.3 ((− 0.9)-1.4)	+ 0.3 ((− 1.5)-2.2)	> 0.950
% median weight change (IQR)	+ 0.7 ((− 3.1)-4.0)	+ 0.2 ((− 0.9)-1.8)	+ 0.2 ((− 2.0)-2.4)	> 0.950
Side effects				
Any side effects, *n* (%)	12 (66.7)	15 (83.3)	27 (75.0)	0.443
Irregular bleeding, *n* (%)	9 (50.0)	15 (83.3)	24 (66.7)	0.075
Nausea/bloating, *n* (%)	3 (16.7)	2 (11.1)	5 (13.9)	> 0.950
Abdominal discomfort, *n* (%)	1 (5.6)	3 (16.7)	4 (11.1)	0.603
Mood issues, *n* (%)	2 (11.1)	1 (5.6)	3 (8.3)	> 0.950
Others	Water retention (1)	Breast tenderness (1)		

Continuous variables were summarized as medians and IQR. Categorical variables were presented as frequencies and percentages

Abbreviations: *IQR*, interquartile range; *MA*, megestrol acetate; *LNG-IUS*, levonorgestrel-intrauterine system; *SD*, standard deviation

The overall 3-month CR was 50.0%. The 3-month regression rate was higher in the LNG-IUS arm (61.1% compared to 38.9%). The difference was not statistically significant.

The overall 6-month CR and 9-month CR were 80.6% and 88.9% respectively. The difference between the MA and LNG-IUS arms were not statically significant.

In the MA arm, 1/18 patients (5.6%) had PH at 9 months and 1/18 (5.6%) had PD. In the LNG-IUS arm, 2/18 (11.1%) had PH at 9 months.

### Secondary endpoint — weight gain, side effects

The side effects experienced by patients are summarised in Table 2.

Overall, the patients had a median weight gain of 0.3 kg after treatment. There was no difference in amount of weight gain, or percentage weight gain in either treatment arm.

Twenty-seven out of 36 patients (75.0%) experienced side effects. Side effects were more common in the LNG-IUS arm compared to the MA arm (83.3% vs. 66.7%). Irregular bleeding and abdominal discomfort were more common in the LNG-IUS arm, while nausea or bloating and mood issues were more common in the MA arm. Overall, there was no statistical difference between side effects experienced in either arm.

### Secondary endpoint — patient tolerability

One patient in the MA arm exited the study and was switched to LNG-IUS. She underwent 3 months of MA. Her pre-operative blood tests before her follow-up D&C showed polycythaemia and transaminitis. She was referred to haematology for review. The risks and benefits of continuing MA were discussed, and the patient was keen to switch her treatment to LNG-IUS. Her transaminitis resolved after the cessation of MA.

### Secondary endpoint — recurrence and fertility outcomes

The recurrence and fertility outcomes were assessed in the 32 patients who achieved CR during the trial. The median follow-up period after the initiation of treatment for this group of patients was 22.5 months (range, 4–41 months). At the time of last follow-up, 2 patients were lost to follow-up. One patient opted for hysterectomy because of fibroids, and because she no longer desired fertility conservation.

Among the remaining 29 patients, there were 5 patients who experienced a recurrence of endometrial hyperplasia during the follow-up. The median disease-free interval before recurrence was 23.0 months (range, 21–32 months).

Among the 32 patients with CR, 19 patients were actively trying to conceive after disease regression. The fertility outcomes in these patients are summarised in Table 3. There were 8 pregnancies achieved (6 in the MA arm and 2 in the LNG-IUS arm) and 4 live births (3 in the MA arm and 1 in the LNG-IUS arm). The miscarriage incidence was 50% in our population, with 2 out of the 4 miscarriages presenting as preterm premature rupture of membrane (PPROM) in the second trimester.

**Table 3:** Fertility outcomes among patients with complete regression at 9 months

	MA (N = 18)	LNG-IUS (N = 18)	Overall (N = 36)
Fertility outcomes			
Regression, *n*	16	16	32
TTC, *n*	11	8	19
IVF	3 patients underwent IVF	3 patients underwent IVF	6
Pregnancies	6 pregnancies [3 spontaneous, 1 LTZ OI, 2 IVF]	2 pregnancies [1 spontaneous, 1 IVF]	8
LBR, *n*	3	1	4
Miscarriage, *n*	1 × PPROM/ chorioamnionitis at 23/52 2 × 1st trimester miscarriage	1 × PPROM at 19/52, E Coli UTI	4

Abbreviations: *MA*, megestrol acetate; *LNG-IUS*, levonorgestrel-intrauterine system; *TTC*, trying to conceive; *IVF*, in vitro fertilisation; *LTZ OI*, letrozole ovulation induction; *LBR*, live birth rate; *PPROM*, preterm premature rupture of membrane; *UTI*, urinary tract infection

### Secondary endpoint — persistent endometrial hyperplasia at 9 months

There was one patient in the MA arm and one patient in the LNG-IUS with persistent endometrial hyperplasia at 9 months of treatment. These patients declined hysterectomy and opted to continue medical treatment.

The patient in the MA arm declined add-on LNG-IUS. She remained on MA and her last biopsy showed partial response (endometrial hyperplasia without atypia). She was still not keen for hysterectomy or LNG-IUS.

The patient in the LNG-IUS arm declined add-on MA. She remained on LNG-IUS and achieved CR at 15 months of treatment. She underwent one cycle of in vitro fertilisation (IVF) but was unsuccessful.

### Secondary endpoint — progression to cancer

One patient in the MA arm had progressive disease to EAC after 3 months of treatment. She was referred to the gynaecologic oncology unit and underwent a multidisciplinary discussion. She was continued on MA with LNG-IUS add-on. She achieved complete regression after 10 months of dual therapy. She underwent two cycles of IVF. Her second thaw cycle resulted in a first trimester missed miscarriage.

## Discussion

### Main findings

LNG-IUS was non-inferior to MA for the treatment of AEH. There were no significant differences in 3-month, 6-month and 9-month CR between both arms. The 9-month CR rate was 88.9%, which was comparable to previous studies [6, 8, 9]. There is a trend towards faster regression with LNG-IUS but the study was not powered to assess for this outcome. No difference was found in recurrence or pregnancy rates between the two arms.

### Strength and limitations

There are limited prospective trials comparing the regression outcomes of MA to LNG-IUS in the treatment of AEH. There is even less prospective data on fertility outcomes in this group of patients. To our knowledge, this is the second RCT on the topic with a previous publication by Xu et al. in 2023 [8]. The strength of the study is the randomised controlled design over three institutions. The small sample size allowed for close adherence to trial protocol and follow-up, and the drop-out rate was low (5.0%).

However, the major limitation of the study, however, is the small sample size, partly contributed by recruitment limitations during the COVID-19 pandemic. As such, the sample size did not reach the statistical power to confirm non-inferiority of LNG-IUS to MA. Moreover, the follow-up time after complete regression was relatively short and a longer follow-up would have allowed more accurate analysis on the rates of recurrence, pregnancy, and live birth.

Nonetheless, in spite of the limitations above, the regression and fertility outcomes from this trial can be included in future meta-analyses to expand high quality data on this topic.

### Interpretation

LNG-IUS and MA were equally effective in resulting in CR by 9 months (88.9%). LNG-IUS may be associated with earlier regression. The patients in LNG-IUS arm had a higher 3-month CR compared to MA (61.1% vs. 38.9%), although this was not statistically significant. The recent RCT by Xu et al. had similar findings [8]. The Kaplan–Meier estimate of 16-week CR rates in their study were higher in the LNG-IUS group (35.0%) compared with the MA group (19.2%), although the difference was not significant. The difference was less prominent at 32 weeks with the 32-week CR rates of 72.0% and 63.1% for LNG-IUS and MA respectively. The CR rates were lower than in our study population. This may be in part contributed by the higher compliance and lower drop-out rate in our study population compared to the study population in Xu et al.

Side effects of patients in both arms were similar. One patient in our study opted to change from MA to LNG-IUS in view of transaminitis. Serious side effects such as thromboembolic events or breast cancer were not experienced by any of our patients. Reassuringly, none of the patients in the study by Xu et al. experienced these serious adverse events either [8].

Obesity is strongly associated with the development of AEH [4]. The RCT by Xu et al. showed that MA was associated with a statistically significant association with weight gain [8]. In their study, 43.1% in the MA arm had Grade 3 weight gain, compared to 8.3% in the LNG-IUS arm. This was not reproduced in our study. The median weight gain was minimal in both arms. This may be in part due to study population difference. The mean BMI was 25.0 kg/m^2^ in Xu et al. compared to the median BMI of 34.5 kg/m^2^ in our study. As our study population had a high incidence of obese patients, there was an emphasis on the importance weight control for all patients enrolled in the study, and this may account for minimal weight gain during the study. About 1 in 3 patients in our study had concomitant diabetes mellitus or impaired glucose tolerance or impaired fasting glucose. Hence, it is important to screen for metabolic diseases in patients with AEH. The 2-h oral glucose tolerance test screening should be the choice of screening to diagnose impaired glucose tolerance and impaired fasting glucose in order to adequately manage the patient’s comorbidities for long-term health and fertility optimisation. Fasting insulin has also been used as a screening modality for metabolic syndrome, and metformin has been used as an adjunct for treatment of endometrial hyperplasia [11, 15]. However, the efficacy of metformin for treatment of endometrial hyperplasia and the ideal target group have yet to be well established.

The fertility outcomes for the LNG-IUS and MA arms in our study were similar. Earlier regression in the LNG-IUS arm did not give the patients an advantage in live birth outcomes. We do note however, this is limited by the small number in the trial, and the relative short follow-up post complete regression. In Xu et al. [8], the 1-year pregnancy rate in the LNG-IUS arm was 37.5% and 40.7% in the MA arm. The pregnancy rate in our study group was slightly lower at 31.6%. In our study population, the rate of miscarriage was 50% which is higher than the baseline risk. Xu et al. reported 36 women with successful pregnancies, resulting in 18 live births (50%), 12 miscarriages (33.3%) and 6 ongoing pregnancies (16.7%). These numbers, while small, amount to some reassurance that successful pregnancy outcomes can be achieved after regression of AEH. Nonetheless, the data may also suggest that miscarriage and PPROM may be higher in this group of patients and caution should be taken. The miscarriage and PPROM risk may be contributed in part by the selection bias of a higher risk population (obesity, insulin resistance, polycystic ovarian syndrome) as well as contributory factors from the disease and treatment. The endometrium may be affected by the disease pathology and/or multiple D&C, with addition of concomitant chronic endometritis. The patients are also at higher risk of cervical incompetence from repeated cervical instrumentation. More studies with long-term outcomes are needed to ascertain this correlation. Meanwhile, it is prudent to have closer antenatal follow-up for these patients who conceive after treatment of AEH.

## Conclusion

Our trial showed overall high regression rates of AEH at 9 months (88.9%) in patients treated with either MA or LNG-IUS. The patients in LNG-IUS arm had a higher 3-month CR compared to MA (61.1% vs. 38.9%), although the 3-month CR, 6-month CR and 9-month CR were not statistically different between both arms. The side effect profile was comparable between LNG-IUS and MA. Weight gain was not significant in either arm. After complete regression, patients can achieve successful pregnancies. However, patients who conceive after AEH treatment may have higher risk of adverse pregnancy outcomes. More studies with long-term outcomes are needed to ascertain this correlation.

## Data Availability

The data that support the findings of this study are available from the corresponding author, CG, upon reasonable request.
